# Improving Rutting Resistance of Pavement Structures Using Geosynthetics: An Overview

**DOI:** 10.1155/2014/764218

**Published:** 2014-01-08

**Authors:** Sina Mirzapour Mounes, Mohamed Rehan Karim, Ali Khodaii, Mohammad Hadi Almasi

**Affiliations:** ^1^Centre for Transportation Research, Faculty of Engineering, University of Malaya, 50603 Kuala Lumpur, Malaysia; ^2^Department of Civil Engineering, Amirkabir University of Technology, 158754413 Tehran, Iran

## Abstract

A pavement structure consists of several layers for the primary purpose of transmitting and distributing traffic loads to the subgrade. Rutting is one form of pavement distresses that may influence the performance of road pavements. Geosynthetics is one type of synthetic materials utilized for improving the performance of pavements against rutting. Various studies have been conducted on using different geosynthetic materials in pavement structures by different researchers. One of the practices is a reinforcing material in asphalt pavements. This paper intends to present and discuss the discoveries from some of the studies on utilizing geosynthetics in flexible pavements as reinforcement against permanent deformation (rutting).

## 1. Introduction

Over the performance life period of pavement structure, it is vulnerable to different kinds of distresses. Permanent deformation (rutting) is one of the serious distresses in which pavement structure may be involved. A lot of research has been conducted so as to prevent diminishing pavements by rutting phenomenon. Both traditional and modern methods have been taken as measures to deal with such distress. One of the latter methods is related to reinforcing pavement structures by means of geosynthetics. Using geosynthetic materials as a reinforcing means in pavement structure mostly in road base and embankment is well investigated, and many researches on reinforcement of asphalt concrete are involved in prevention of reflection cracking [1, 2]. However, very little research is performed on the influence of reinforced asphalt concrete on the formation of plastic and shear strains in asphalt concrete [3]. In this paper, attempt has been made to review some of the reported effects of geosynthetics on rutting in pavement structures.

## 2. Pavements

Flexible pavements generally consist of a prepared roadbed underlying layers of subbase, base, and surface courses [4]. Surface courses are usually called asphalt concrete which is a type of material which can be produced by compaction of a mixture consisting of crushed rock or gravel, sand or crushed stone, filler, and bitumen, from certain amount of each. It can only attain the required physical and mechanical qualities after compacting. Asphalt concrete may have different physical existence called plastic, viscoelastic, and elastic under different environmental conditions. Rheology which is a science about the fluidity of materials can give the most complete and accurate description of the asphalt concrete operation [5]. Over the life period of asphalt concrete in pavement structure, it is vulnerable to different kinds of distresses mostly known as fatigue cracking, rutting, and thermal cracking.

## 3. Geosynthetics

Geosynthetic is a planar product manufactured from a variety of synthetic polymer materials that are specifically fabricated to be used in geotechnical, geoenvironmental, hydraulic, and transportation engineering related materials as an integral part of a man-made project, structure, or system [6]. They are usually composed of seven main categories called geotextiles, geogrids, geonets, geomembranes, geosynthetic clay liners, geofoam, and geocomposites. When the target is reinforcing soil and asphalt pavement, out of the above-mentioned seven categories, geotextiles, geogrids, and geocomposites are the ones usually being practiced [7]. The most important functions of geosynthetic materials concerned with transportation engineering are separation, reinforcement, filtration, drainage, and acting as a liquid barrier [8], but in the asphalt layer if properly installed they mainly function as fluid barrier, cushion, and reinforcement.

## 4. Permanent Deformation (Rutting)

**Figure 1:**
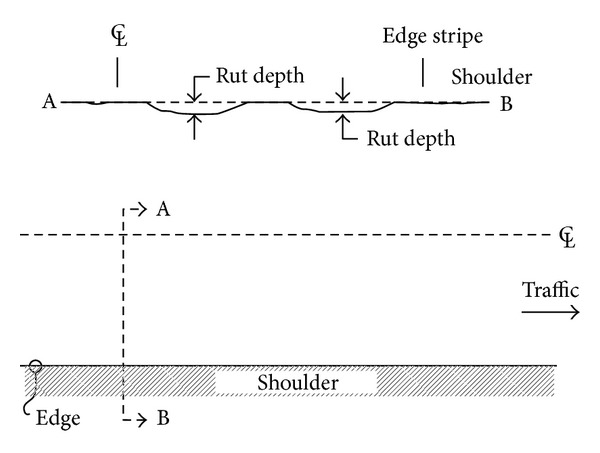
Schematic representation of rutting [11].

**Figure 2:**
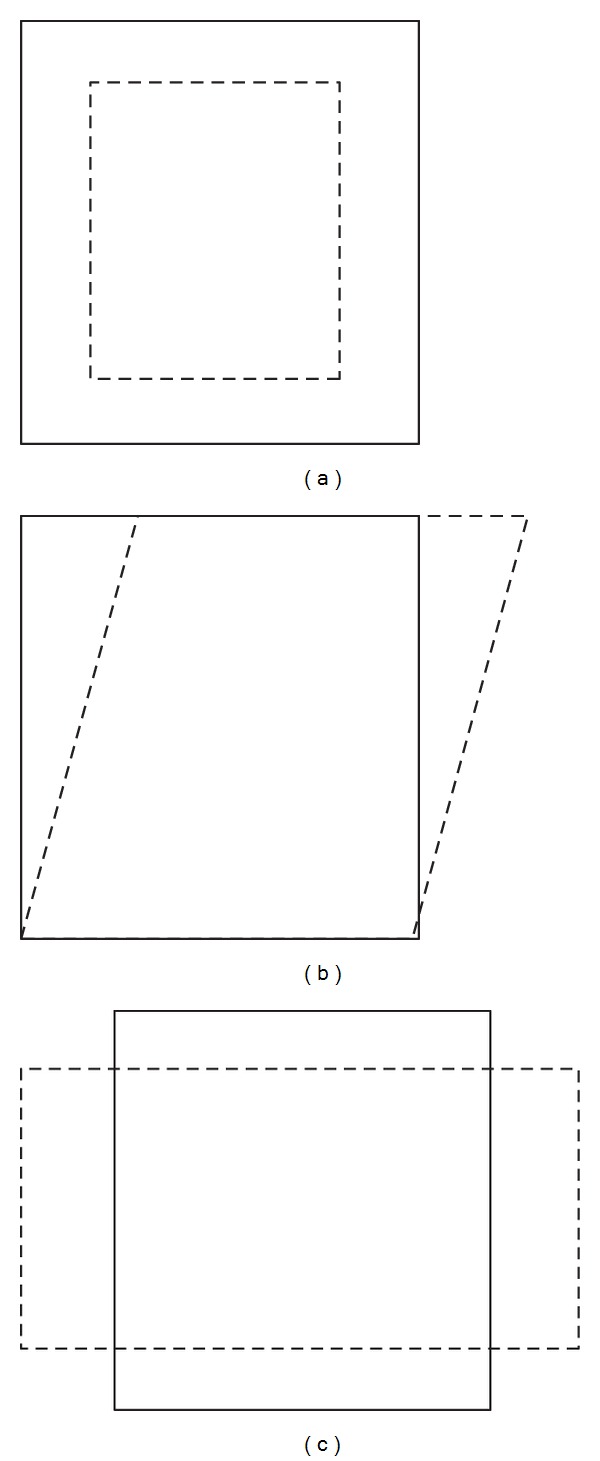
Schematic illustration of volume change (a) and shape distortion (b, c) [10].

Permanent deformation or rutting of asphalt reveals itself as depressions which are formed in the pavement's wheel path (Figure 1). Water collects in these depressions and cannot drain freely off the pavement surface. This could cause aquaplaning and therefore rutting is also a potential safety hazard. Generally, rutting development in asphalt layers can be described as a two-stage process, namely, consolidation (densification) which is concerned with volume change of asphalt layer and shape distortion [9, 10].  Figure 2 shows schematically both forms of deformations.

Rutting resistance of a paving asphalt mixture is one of the important considerations in asphalt mix design, as a large part of accumulated rutting in pavement structure occurs in the surface layer. Formation of ruts, having started in the initial stage of pavement operation, increases with the growth of the flow of heavy traffic. The main cause of rut initiation is shear strains in asphalt. There are several causes of such deformations. Some of them are high temperature, unsuitable mixture, and traffic loads [12]. In general, rutting at higher temperatures occurs due to pavement consolidation and/or HMA experiences lateral movement which is a shear failure [13]. Moreover, analyzing the rutting behaviour of composite pavements in the State of Louisiana depicted that cumulative ESAL, thickness of the portland cement concrete layer, highway functional classification, and surface age can highly influence permanent deformation [14].

## 5. Asphalt Concrete Reinforcement

Reinforcing is a structural measure of increasing strength against the variety of stresses and improving its strength characteristics. It refers to mobilizing stresses in some layers, mostly in geosynthetics. Geosynthetic reinforcement of pavement leads to changing in rheological model of asphalt pavements [5, 15]. Several studies have been conducted in order to investigate the effect of geosynthetic materials on asphaltic pavements. In a research done by Laurinavicius and Oginskas, on testing sections with equal asphalt concrete layer thickness of reinforced and control ones, modulus of elasticity and rut depth are measured in different seasons. It was shown that rutting depth depends on modulus of elasticity of asphalt concrete. In its turn, the modulus of elasticity of asphalt concrete is dependent on the type of geosynthetic material used. Thus, it is worthwhile to use geogrid to improve the strength properties of asphalt concrete and reduce the shear strain. In other words, rutting depth depends on the type of geosynthetic material which is used [3].

In a research by Jenkins et al., improvement was observed in rutting behavior of geogrid reinforced samples compared with unreinforced ones. Moreover, geogrids with smaller aperture size performed better under rutting test [16]. Monotonic, cyclic, and dynamic loading were adopted to study geosynthetic reinforced asphalt pavement by Ling and Liu. It was observed that the stiffness and bearing capacity of the asphalt concrete pavement were increased in presence of geogrid. The geogrid stiffness and its interlocking with the asphalt concrete contributed to the restraining effect. The developed strains in the geogrid around the vicinity of the loading area manifested the restraining effect of geogrid. Moreover, there was a reduction in settlement over the loading area of reinforced pavement compared with that of unreinforced pavement. The improvement was more significant for dynamic loading compared with that for static loading [17].

In another research conducted by Bertuliene et al., rut depth was measured on experimental road section from the day of section's construction and the geosynthetic-reinforced and control sections were compared. It was shown in theoretical research that pavement ruts can be affected by geosynthetic materials related to shear deformation in asphalt pavement. Experimental research also showed that there is a positive effect on formation and development of ruts by insertion of geosynthetic materials. For instance, rut depth on the road sections reinforced with geosynthetic materials is 1.4 times lower than that of unreinforced section [12]. Furthermore, in 1998, the resistance of geogrid-reinforced asphalt concrete against plastic flow and cracks was investigated by means of wheel tracking test. Remarkable increase in durability was reported comparing geogrid reinforced and control samples. Thereby, viscosity increase of asphalt concrete was observed. The crack resistance was strongly correlated to the plastic flow resistance. Reduction in geogrid-mesh size and stronger adhesion of geogrid to the asphalt concrete lead to more improvement in durability. As a result, 10 and 30 times increase in crack resistance and plastic flow resistance, respectively, were achieved in reinforced samples compared to those of control ones. In order to determine the relationship between the results of the wheel tracking test and the real durability of the field, a prototype geogrid embedded at a certain highway and the reinforcement effect were observed for 5 years. Little cracks and rutting were produced in reinforced sections in contrast to the unreinforced sections [18].

In a study the reinforced and control specimens were subjected to monotonic loadings with load ratios of 0.2, 0.4, 0.8, 1.0, and 1.2. For load ratios of 0.2 and 0.4, rut depth in reinforced specimens is reported 40% less than that of unreinforced specimens. At load ratios above 0.4, some embedded samples sustained more than twice the deformation of unreinforced specimens; however, they withstood over 100 times the number of cycles before terminal cracking. It was also observed that the samples, geogrid of which is embedded in middepth, performed better than those of which the geogrid is applied at the bottom of asphalt layer [19].

## 6. Granular Layer Reinforcement

**Figure 3:**
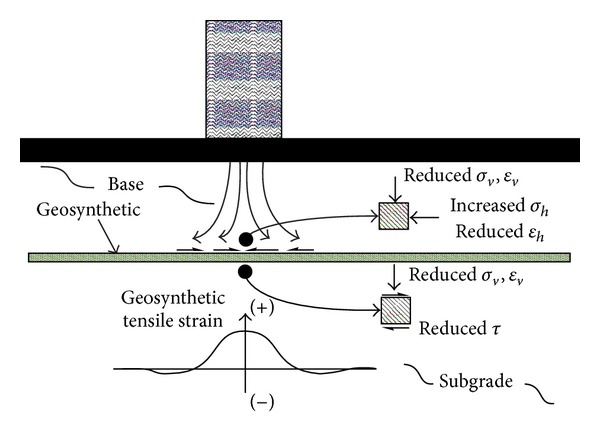
Schematic illustration of base reinforcement mechanism [21].

In granular material layers, the mechanism of rut depth reduction through geosynthetic reinforcement may be explained as follows:

Lateral movements are prevented by aggregate confinement, leading to increase in bulk stress, and aggregate layer stiffness, along with decrease in vertical stress on top of subgrade and vertical compressive strain reduction in lower half of base and in the subgrade [20]. The mechanism of base layer reinforcement is represented in Figure 3.

Over the period of pavement construction, there are usually two feasible alternatives for ground improvement, namely, soil stabilization and geosynthetic reinforcement. At times, some of the contractors prefer to use geosynthetics to reinforce subgrade [22].

In a research by Tang et al., mechanical and physical properties of geogrids are identified by index testing, bench-scale testing, and accelerated traffic loading. Such properties of geogrid are critical to its efficiency in subgrade stabilization. Surface rutting at various trafficking stages was taken as a measure to evaluate pavement section performance. Based on this study, aperture size, tensile strength at small strains, junction strength, and flexural rigidity are identified as the most important attributes of geogrids in pavement subgrade stabilization. After excluding the effect of air void variation of asphalt concrete which appears having some influence on results, substantial benefits of geogrid stabilization for weak subgrade soil can be observed. In a comparison of rutting behavior using accelerated pavement tests among sections, certain geogrids are recognized to be more suitable to be used for stronger subgrade. So, it may be claimed that adequate reinforcement for weak subgrades will be supplied by geogrids that fulfill the criteria in physical and mechanical properties recognized [23].

Montanelli et al. inferred that applying geogrid between base course and subgrade can lead to more uniform load distribution in pavement structure. Also, settlement at the asphalt-aggregate and aggregate-subgrade interfaces can be reduced, and it was shown that geogrid reinforcement is a cost-effective solution for flexible pavement system [24]. It is also reported that percent reduction of rutting would increase by subgrade CBR reduction, between reinforced and unreinforced sections of an in-ground experiment [25]. However, care should be taken in embedding methods of geosynthetics. In a study by Han et al. in which different types of geotextiles were placed within a base course, the numerical analysis for geotextile-reinforced bases showed that rutting would increase and advantageous effects of geotextile confinement would be minimized by any possible slippage at the interface of the geotextile [26].

In another effort by Zhao and Foxworthy, laboratory, nondestructive, and full-scale in-ground testing were utilized and it was shown that geogrid can lead to significant decrease in rutting. Moreover, the cost benefits of reinforced pavements related to reduced materials and construction costs are also investigated, and it is concluded that not only material cost savings but also improved workability for the construction platform over low CBR subgrade can be addressed by utilizing geogrid [27].

In terms of temporary unpaved roads, significant rutting depth, for example, 50–100 mm, is often acceptable. However, a deep permanent deformation in subgrade can lead to contamination of base course with subgrade soil. Thereby, it may necessitate a replacement of base course [28]. In order to encounter with such issues in unpaved roads, geosynthetic reinforcement, particularly geogrid reinforcement of base course, can be very useful. Reinforcement of base course materials can prevent the lateral movement and improvement of compression and flexural stiffness of base course leading to reduction in surface rutting, vertical strains within the base course, better distribution of traffic loads, and reduction in maximum vertical stress on the subgrade. Also, by reinforcement of base course, the transmitted shear stress from base course to subgrade can be reduced resulting in improvement in bearing capacity of subgrade, and tensioned membrane support can be provided where deep rutting occurs [15, 29, 30].

Another laboratory test on unpaved roads using equivalent standard axle load under stress controlled environment and application of cyclic loads, well demonstrated the reinforcement effect of geotextile through membrane action. It was also found that the reinforcement effect increases by permanent deformation until the grab strength of geotextile [31]. Reduction in the rutting as a function of the trafficking in unpaved roads was also reported by Dewangan et al. [32]. It was also shown that base layer thickness can be reduced up to 20% by reinforcement [33]. However, in terms of thick pavements mixed results were obtained [34].

Some additional potential benefits to base course provided by reinforcement can be as the following [28]:

- prevention of shear failure within the base course,
- tensioned membrane direct support of traffic load after significant rutting where traffic is channelized,
- prevention of tension cracking at the bottom of the base course, which minimizes contamination of the base course material with subgrade soil as the layer flexes under load,
- prevention of loss of base course aggregate into soft subgrade soil.

In a research conducted by Retzlaff et al., it has been reported that geogrid can cause an increase of 40% and 30%, in bearing capacity and rutting resistance of unbound material layers in road construction, respectively [35]. Retzlaff and Voskamp also stated that reinforced subbase layer density can crucially affect the reinforcement mechanism, and associated elongation in geogrid is correlated to density of the aggregate. Nevertheless, after analysis it was deduced that, only from rutting itself, no clear estimate can be made about the caused elongation in the geogrid. Thereby, it may be inferred that geogrid laid on top of lower bearing capacity subgrades will experience larger elongations [36].

## 7. Conclusion

Flexible pavements generally consist of a prepared roadbed underlying layers of subbase, base, and surface courses. Surface layer is asphalt concrete which is a type of material which can be produced by compaction of a mixture consisting of crushed rock or gravel, sand or crushed stone, filler, and bitumen, from certain amount of each. Over the life period of asphalt concrete in pavement structure, it is vulnerable to different kinds of distresses mostly known as fatigue cracking, rutting, and thermal cracking. A lot of research has been conducted so as to prevent diminishing pavements by rutting phenomenon. One of the prevention methods is geosynthetic reinforcement of pavements.

Based on investigation of different studies in this paper in terms of asphalt concrete reinforcement, it appears that geosynthetic reinforcement particularly some certain geogrids positively influences permanent deformation of asphaltic pavements. This influence was stronger in geogrid reinforced samples when the laid location of geogrid was in middepth of asphalt concrete compared to embedding at the bottom. It was observed that geosynthtic reinforcement leads to an increase in elastic modulus of asphalt concrete that rut depth depends on. In its turn, the modulus of elasticity of asphalt concrete is dependent on the type of geosynthetic material used. Moreover, there was an improvement in durability (plastic flow resistance and crack resistance) of asphalt concrete. Mesh size and adhesion of geogrid to asphalt concrete played important roles in durability improvement.

Regarding geosynthetic reinforcement of granular material layers, aperture size, tensile strength at small strains, junction strength, and flexural rigidity of geogrids are recognized as the most important attributes in pavement subgrade stabilization. Furthermore, the laying down location of geogrid in pavement structure is found to be important. It was also shown that in order to avoid deep permanent deformation on temporary unpaved roads due to reasons such as lateral movement of base course and transmitting of shear stress from base course to subgrade, geosynthetic reinforcement can be effective. However, reinforcement effects can be appeared in case of properly constructed pavement structures. For instance, slippage at the interface of geotextile would increase rutting and reduce confinement effect.

## References

[1] Kim KW, Doh YS, Lim S (1999). Mode I reflection cracking resistance of strengthened asphalt concretes. *Construction and Building Materials*.

[2] Cleveland GS, Button JW, Lytton RL (2002). *Geosynthetics in Flexible and Rigid Pavement Overlay Systems to Reduce Reflection Cracking*.

[3] Laurinavičius A, Oginskas R (2006). Experimental research on the development of rutting in asphalt concrete pavements reinforced with geosynthetic materials. *Journal of Civil Engineering and Management*.

[4] (1993). *AASHTO Guide for Design of Pavement Structures*.

[5] Huang YH Pavement analysis and design.

[6] ASTM

[7] Hosseini HRA, Darban AK, Fakhri K (2009). The effect of geosynthetic reinforcement on the damage propagation rate of asphalt pavements. *Scientia Iranica*.

[8] Khodaii A, Fallah S, Moghadas Nejad F (2009). Effects of geosynthetics on reduction of reflection cracking in asphalt overlays. *Geotextiles and Geomembranes*.

[9] Verhaeghe B, Myburgh P, Denneman E Asphalt rutting and its prevention.

[10] Kim YR (2009). *Modeling of Asphalt Concrete*.

[11] Miller JS, Bellinger WY (2003). *Distress Identification Manual for the LTPP*.

[12] Bertuliene L, Oginskas R, Bulevicius M Research of rut depth in asphalt pavements reinforced with geosynthetic materials.

[13] Kim Y, Park T-S (2013). Reinforcement of recycled foamed asphalt using short polypropylene fibers. *Advances in Materials Science and Engineering*.

[14] Nur MA, Khattak MJ, Bhuyan MR-U-K (2013). *Rutting Model for HMA Overlay Treatment of Composite Pavements*.

[15] Perkins SW Geosynthetic reinforcement of flexible pavements: laboratory based pavement test sections.

[16] Jenkins KJ, Dennison P, Ebels LJ, Mullins LS 3-D polymer grid reinforcement of asphalt for rut resistance.

[17] Ling HI, Liu Z (2001). Performance of geosynthetic-reinforced asphalt pavements. *Journal of Geotechnical and Geoenvironmental Engineering*.

[18] Komatsu T, Kikuta H, Tuji Y, Muramatsu E (1998). Durability assessment of geogrid-reinforced asphalt concrete. *Geotextiles and Geomembranes*.

[19] Sobhan K, Genduso M, Tandon V Effects of geosynthetic reinforcement on the propagation of reflection cracking and accumulation of permanent deformation in asphalt overlays.

[20] Perkins SW, Edens MQ (2003). A design model for geosynthetic-reinforced pavements. *International Journal of Pavement Engineering*.

[21] Perkins SW (2001). Mechanistic-empirical modeling and design model development of geosynthetic reinforced flexible pavements. *Final Report*.

[22] Foye KC (2011). Use of reclaimed asphalt pavement in conjunction with ground improvement: a case history. *Advances in Civil Engineering*.

[23] Tang X, Chehab GR, Palomino A (2008). Evaluation of geogrids for stabilising weak pavement subgrade. *International Journal of Pavement Engineering*.

[24] Montanelli F, Zhao A, Rimoldi P Geosynthetic-reinforced pavement system: testing and design.

[25] Cancelli A, Montanelli F In-ground test for geosynthetic reinforced flexible paved roads.

[26] Han J, Zhang Y, Parsons R (2011). Quantifying the influence of geosynthetics on performance of reinforced granular bases in laboratory. *Geotechnical Engineering*.

[27] Zhao A, Foxworthy PT (1999). Geogrid reinforcement of flexible pavements: a practical perspective. *Geotechnical Fabrics Report*.

[28] Giroud JP, Han J (2004). Design method for geogrid-reinforced unpaved roads. I. Development of design method. *Journal of Geotechnical and Geoenvironmental Engineering*.

[29] Giroud J, Ah-Line C, Bonaparte R Design of unpaved roads and trafficked areas with geogrids.

[30] TenCate Geosynthetic reinforcement of the aggregate base/subbase courses of pavement structures.

[31] Bhosale SS, Kambale BR Laboratory study for evaluation of membrane effect of geotextile in unpaved road.

[32] Dewangan A, Gupta DP, Bakshi RK, Manchiryal RK The significance of geotextile in unpaved roads with special reference to stress analysis.

[33] Holder WH, Andreae J Geogrid reinforcement to reduce pavement section thickness: a case study.

[34] Helstrom CL, Humphrey DN, Hayden SA Geogrid reinforced pavement structure in a cold region.

[35] Retzlaff J, Turczynski U, Schwerdt S The effect of geogrids under unbound sub base layers.

[36] Retzlaff J, Voskamp W Comparison of designs based on standard tests with on site measurements of the reinforcement effect of geogrids in executed road projects.

